# Wnt signaling is involved in 6-benzylthioinosine-induced AML cell differentiation

**DOI:** 10.1186/1471-2407-14-886

**Published:** 2014-11-27

**Authors:** Shaolei Zang, Na Liu, Hongchun Wang, David N Wald, Na Shao, Jingru Zhang, Daoxin Ma, Chunyan Ji, William Tse

**Affiliations:** Department of Hematology, Qilu Hospital, Shandong University, 107 West Wenhua Road, Jinan, Shandong 250012 P.R. China; Department of Pathology, Case Western Reserve School of Medicine, Cleveland, Ohio 44106 USA; Mary Babb Randolph Cancer Center, West Virginia University School of Medicine, Morgantown, WV USA

**Keywords:** Wnt, AML, Differentiation, 6-BT

## Abstract

**Background:**

We previously demonstrated that 6-benzylthioinosine (6-BT) could induce the differentiation of a subset of acute myeloid leukemia (AML) cell lines and primary AML cells regardless of their cytogenetics. In this study we investigated whether Wnt signaling pathways played roles in 6-BT-induced differentiation of AML cells.

**Methods:**

We induced differentiation of HL-60 leukemic cells and primary AML cells in vitro using 6-BT. Real-time PCR (qPCR), western blot, and luciferase assays were used to examine the molecules’ expression and biological activity in canonical and noncanonical Wnt signaling pathways. AML cell differentiation was measured by the Nitroblue tetrozolium (NBT) reduction assay.

**Results:**

6-BT regulated the expression of both canonical and non-canonical Wnt signaling molecules in HL-60 cells. Both 6-BT and all-trans-retinoic-acid (ATRA) reduced canonical Wnt signaling and activated noncanonical Wnt/Ca^2+^ signaling in HL-60 cells. Pre-treatment of HL-60 cells with an inhibitor of glycogen synthase kinase-3β (GSK-3β), which activated canonical Wnt signaling, partly abolished the differentiation of HL-60 cells induced by 6-BT. Pre-treatment of HL-60 cells with an inhibitor of protein kinase C (PKC), resulting in inactivation of non-canonical Wnt/Ca^2+^ signaling, abolished 6-BT-induced differentiation of HL-60 cells. Several molecules in the non-canonical Wnt/Ca^2+^ pathway were detected in bone marrow samples from AML patients, and the expression of *FZD4, FZD5, Wnt5a* and *RHOU* were significantly reduced in newly diagnosed AML samples compared with normal controls.

**Conclusions:**

Both canonical and non-canonical Wnt signaling were involved in 6-BT-induced differentiation of HL-60 cells, and played opposite roles in this process. Wnt signaling could be involved in the pathogenesis of AML not only by regulating self-renewal of hematopoietic stem cells, but also by playing a role in the differentiation of AML cells.

**Electronic supplementary material:**

The online version of this article (doi:10.1186/1471-2407-14-886) contains supplementary material, which is available to authorized users.

## Background

Wnt signaling pathways are highly conserved and regulate cell fate decision at all stages of development in multiple tissue types, including hematopoietic stem cells [1, 2]. Deregulation of canonical or noncanonical Wnt signaling pathway plays critical roles in the pathogenesis of various cancers including AML [3]. However, the mechanisms of co-ordination between these two branches of Wnt signaling pathway in AML cell differentiation are largely unexplored.

In the canonical Wnt/β-catenin signaling pathway, engagement of a Wnt protein by a Frizzled (FZD) receptor leads to stabilization of β-catenin, which then translocates into the nucleus to initiate target gene expression through interaction with the TCF/LEF transcriptional complex [4]. Deregulation of the canonical branch of Wnt signaling pathway by aberrant stabilization and constitutive activation of β-catenin is linked to the initiation and progression of AML and other cancers [5–7]. Most of human AML has up-regulated and nuclear localized β-catenin compared with normal bone marrow CD34^+^ cells [8]. In both AML cell lines and primary samples, silencing genes associated with the canonical Wnt/β-catenin pathway through methylation have been observed [9, 10]. In addition, inhibition of the Wnt/β-catenin pathway by small-molecules results in apoptosis of AML cells [11]. The non-canonical Wnt signaling pathway, independent of β-catenin, is a Ca^2+^-releasing pathway that is activated by the Wnt-stimulated G proteins. Calcium/protein kinase C(PKC) and calmodulin-dependent kinase II (CaMKII) were considered to be primary mediators of this signaling pathway [12, 13]. Non-canonical Wnt binds to an FZD receptor, leading to release of intracellular calcium and activate enzymes, such as PKC and CaMKII. Human Wnt4, Wnt5a, and Wnt11 are ligands for receptors or co-receptors FZD2, FZD5, FZD6 and FZD7. A recent study showed that non-canonical Wnt signaling was also closely related to tumorigenesis [14]. Wnt5a was silenced in an animal model of AML, which suggested that it might act as a tumor suppressor [15].

We have demonstrated that 6-BT induced differentiation of various AML cell lines and primary AML cells [16]. Since the Wnt signaling pathways play a critical role in the differentiation of several types of cells, including osteoblasts, cardiomyocytes and neurons [17], we hypothesized that it might also be involved in the 6-BT-induced differentiation of AML cells. In this study we demonstrated that both canonical and non-canonical Wnt signaling played a critical role in the 6-BT induced differentiation of AML cells.

## Methods

### Cell lines and chemicals

HL-60 cells were cultured in Iscove’s modified Dulbecco’s medium (Invitrogen, Carlsbad, CA) supplemented with 10% fetal bovine serum (FBS, Gibco, Grand Island, NY) and 1% penicillin–streptomycin. ATRA, PMA and NBT were purchased from Sigma(Sigma-Aldrich, St. Louis, USA). 6-BT was kindly provided by the National Cancer Institute Developmental Therapeutics Program. Bisindoylmaleimide (BIM) and BIO were purchased from CalBiochem. Primary antibodies for β-catenin, p-PKC (Thr638), PKC, p-CaMKII (Thr286), CaMKII and β-actin were purchased from Abcam. Primary antibodies for p-Rac (Ser71) and Rac were purchased from Cell Signaling Technology.

### Patient samples

Bone marrow mononuclear cells of patients treated at Qilu Hospital (Shandong University, Shandong, P.R. China) were obtained between May 2008 and July 2009. Thirty patients had newly diagnosed AML, twelve had AML in complete remission, and ten were normal controls. Informed consent was obtained from each donor. Procedures for collecting samples were approved by the Drug and Clinical Investigations Ethics Committee of the Faculty of Medicine, Qilu Hospital, Shandong University.

### PCR array

Human Wnt signal RT^2^ Profiler™ PCR array (PASH-043) was generously provided by SuperArray Bioscience Corporation (Frederick, MD). The PCR array was performed according to the manufacturer’s instructions. Briefly, total RNA was isolated from HL-60 cells after treatment with 6-BT (10 μM) or vehicle (0.01% DMSO) for 3 days. Reverse transcription was performed with M-MuLV reverse transcriptase (Fermentas) using an oligo(dT)18 primer. Genomic DNA contamination was eliminated by Dnase treatment using an RNeasy Micro Kit (Qiagen). Expression of Wnt molecules was tested by PCR on ABI Prism 7700 (Applied Biosystems). For data analysis, the ^ΔΔ^Ct method was used. For each gene, fold changes were calculated as the difference in gene expression between 6-BT- or vehicle-treated cells; a positive value indicates gene up-regulation and a negative value indicates gene down-regulation.

### Real-time RT-PCR

Total RNA was isolated from HL-60 cells treated with 6-BT or vehicle for 1 day or 3 days, using TRIzol reagent (Invitrogen). RNA was transcribed into cDNA using the Enhanced Avian RT First Strand Synthesis kit (Sigma). RT-PCR was performed in triplicate using FastStart SYBR Green Master (Roche Diagnostics) on an Applied Biosystems 7500 Fast Real-Time PCR System. Primers used are available upon request.

For patient samples, qRT-PCR was performed using SYBR Green PCR Master Mix (Toyobo) on an ABI Prism 7500 sequence detection system. All reactions were carried out in 20-μl reaction volume in triplicate. Fold changes in gene expression were determined using the 2^-ΔΔ^CT method with β-actin as an endogenous control.

### NBT reduction assay

We used NBT reduction to evaluate differentiation of AML cells. To perform the NBT assay, 100 μL of HL-60 cells (5 × 10^5^ cells/mL) were cultured in 96-well plates. Cells were first treated with 10 μM of 6-BT or DMSO (0.01%) for 5 d, then with 20 uL of a solution of NBT (5 mg/mL) and PMA (100 ng/mL). Cells were incubated at 37°C for 30 min and at least 200 cells were counted for the positive percent.

### Western blot

After treatment with 6-BT or ATRA, HL-60 or primary AML cells were harvested by centrifugation and washed twice with phosphate-buffered saline (PBS), then solubilized in radio immunoprecipitation assay (RIPA) lysis buffer containing 1% Triton X-100, 1% sodium deoxycholate, 0.1% sodium dodecyl sulphate (SDS), 0.15 mol/l NaCl and 0.05 mol/l Tris–HCl, pH 7.2. Protein concentrations were determined with the bicinchoninic acid (BCA) assay protein reagent kit (Sangon) according to a standardized curve. Total proteins (30 ug/lane) were separated by 10% SDS–polyacrylamide gel electrophoresis and transferred onto nitrocellulose membranes using standard procedures. Non-specific sites were blocked with 5% nonfat milk in PBS with 0.1% Tween-20. Primary antibodies were used according to the manufacturer’s instructions. The near-infrared fluorescence-labeled secondary antibodies detecting primary antibodies were IRDye 680 Goat Anti-Rabbit IgG and IRDye 800CW Goat Anti-Mouse IgG (Li-Cor Biosciences, Lincoln, NE). Detection and quantification were performed with the Li-Cor Odyssey imaging system and its software.

### Transient transfection and luciferase assays

The TOPFlASH is a luciferase reporter of β-catenin-mediated transcriptional activation. The backbone of TOPFlASH is the pTA-Luc vector of Clontech, which provides a minimal TA viral promoter driving expression of the firefly luciferase gene. 7 TCF/LEF binding sites were cloned into the Mlu1 site of this vector. The negative control FOPFLASH construct contains mutated TCF/LEF binding sites [18]. NFAT-luciferase construct, which contains NFAT binding sites, is used to determine the activity of the noncanonical Wnt signaling pathway [19]. The TOPFLAH and FOPFLASH and NFAT-luciferase constructs were from Addgene. Renilla luciferase pRL-TK was cotransfected as an internal control for transfection efficiency. Transfections were performed using a Nucleofector (Amaxa) according to the manufacturer’s instructions with minor modifications. Briefly, 1 × 10^6^ HL-60 cells were transfected with 2.5 μg of either TOPFLASH, FOPFLASH or NFAT luciferase along with 0.25 μg pRL-TK. Vehicle (0.01% DMSO), positive control (10 mM LiCL) or 6-BT (10 or 20 μM) were added 24 hours after transfection. After another 24 hours, cell lysates were prepared and reporter activity was measured using the Dual-Luciferase Reporter Assay System (Promega).

### Intracellular Ca^2+^ concentration assays

Cells were washed twice with PBS, then loaded with Fluo-3/AM (Molecular Probes) for 30 min, and warmed to 37°C before flow cytometry analysis using a FACScan (Becton Dickinson).

### Immunofluorescence

Immunofluorescence was performed to identify subcellular localization of β-catenin. Three days after treatment with 6-BT (10 μM) or ATRA (1 μM), HL-60 cells were harvested by centrifugation. Drops of cells were plated on polylysine-coated slides and incubated at room temperature for 25 min, then fixed with 4% polyoxymethylene. Cells were permeabilized with 0.5% Triton X-100 in PBS for 15 min, and then blocking was carried out with goat serum for 30 min to minimize nonspecific binding of the primary antibody. The β-catenin antibody (ab2982, Abcam, Cambridge, MA) was applied at a 1:100 dilution overnight followed by three 5-min washes in PBS. FITC anti-rabit IgG (Jackson Lab) were used to detect β-catenin. Images were captured using a Zeiss Microscopy LSM 780 fluorescent microscope and analyzed with Image J software.

### Statistical analysis

Values are mean ± standard deviation (SD) from 3 independent experiments. Groups were compared using a Student’s two-tailed unpaired t test. For patient samples, the copy number of each gene is presented quantitatively as mean ± SD. The difference in copy number of each gene in the AML-ND, AML-CR, and CON groups was performed using a one-way ANOVA test. SPSS software (version 15.0) was used for all statistical analysis. Tests for statistical significance were two-sided. P values less than 0.05 were considered to indicate statistical significance.

## Results

### The canonical and noncanonical Wnt signaling pathways are differentially regulated upon 6-BT treatment

**Figure 1:**
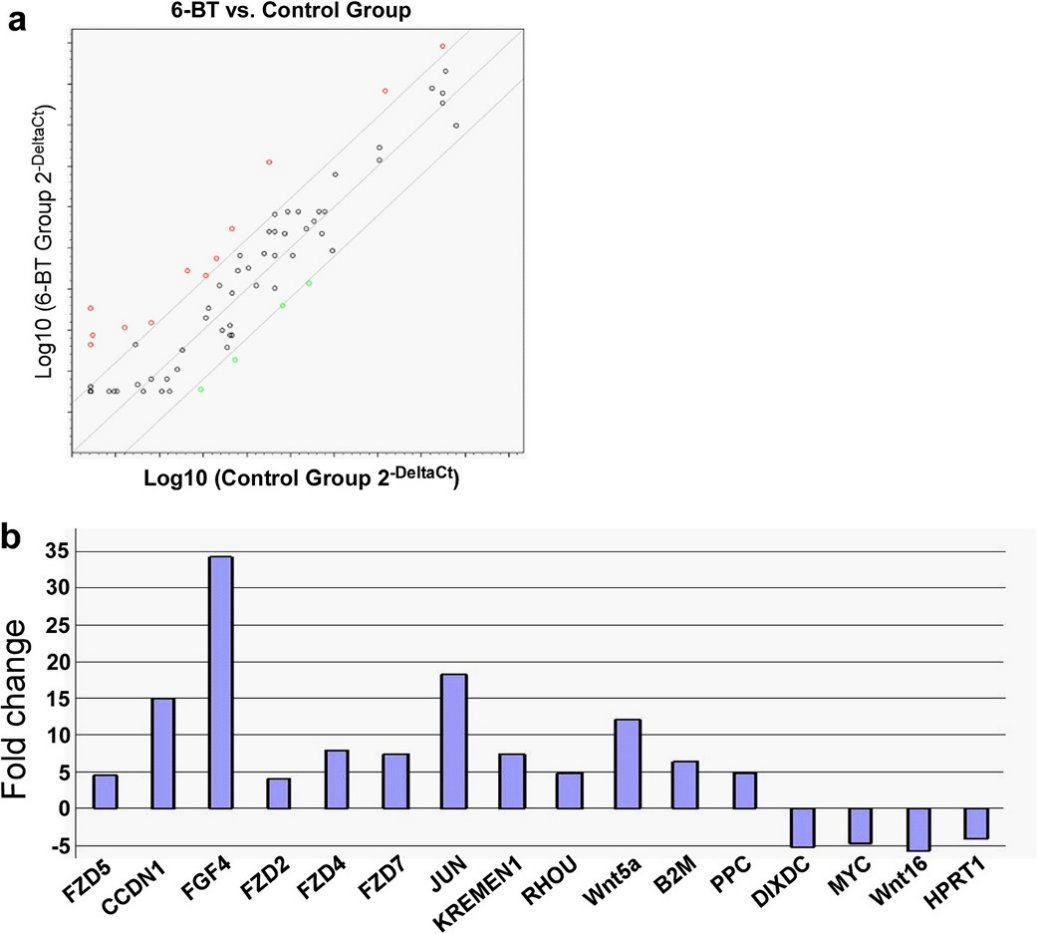
**Wnt signal molecules are regulated upon 6-BT treatment. a**. Scatter plot from Wnt signal RT^2^ Profiler™ PCR array. Red circles represent genes that are up-regulated more than 4-fold upon 6-BT treatment; green circles represent genes that are down-regulated more than 4-fold. **b**. Genes up- or down-regulated more than 4-fold upon 6-BT treatment.

To examine the molecular alterations associated with the 6-BT-induced differentiation of AML cells, we compared transcription of Wnt molecules in HL-60 cells before and after treatment with 6-BT (10 μM) or vehicle (0.01% DMSO) for 3 days. A total of 96 genes, including 5 housekeeping genes, were examined in the RT^2^ Profiler™ qPCR array. Twelve genes, *Wnt5a*, *Wnt11, FZD2*, *FZD4*, *FZD5*, *FZD7*, *JUN*, *KREMEN1*, *RHOU*, *CCND1*, *PPC* and *B2M,* were up-regulated more than 4-fold upon 6-BT treatment (Figure 1a). Four other genes, *Wnt6*, *MYC, DIXDC* and *HPRT1*, were down-regulated more than 4-fold upon 6-BT treatment. Most up-regulated genes (*Wnt5a, FZD2, FZD4, FZD5, FZD7, RHOU)* are Wnt molecules or positive regulators, whereas most down-regulated genes (*Wnt6, MYC, DIXDC*) are in the canonical Wnt signaling pathway (Figure 1b).

### 6-BT increases the expression of noncanonical Wnt signaling molecules while decreases canonical Wnt signaling molecules

**Figure 2:**
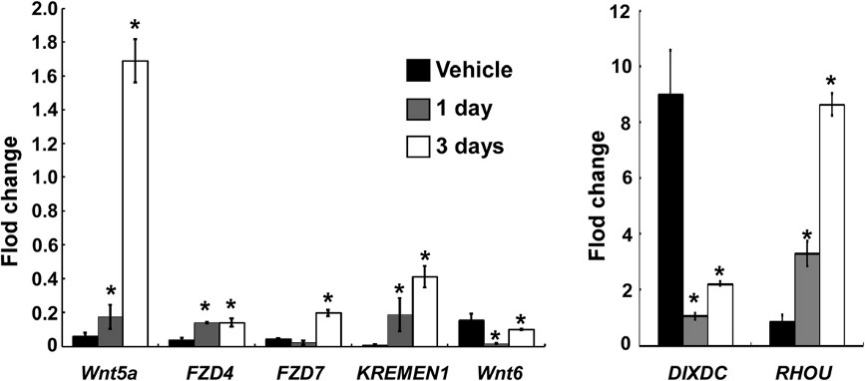
**Transcriptional change of certain Wnt molecules upon 6-BT treatment.** Real-time RT-PCR confirmed that transcription of *Wnt5a*, *FZD4*, *FZD7*, *KREMEN1* and *RHOU* was significantly up-regulated upon 6-BT treatment, while transcription of *Wnt6* and *DIXDC* was significantly down-regulated. Data are from three independent experiments.

We used qPCR to independently verify transcript levels of Wnt genes identified by the PCR array. Transcription of *Wnt5a*, *FZD4*, *FZD7*, *KREMEN1*, *RHOU*, *Wnt6*, and *DIXDC* was compared in HL-60 cells treated with 6-BT or vehicle for 1 day or 3 days. We demonstrated that expression levels of *Wnt5a*, *FZD4*, *FZD7*, *KREMEN1*, and *RHOU* were significantly up-regulated after 6-BT treatment, whereas expression levels of *Wnt6* and *DIXDC* were significantly down-regulated (Figure 2, P <0.05). These results were consistent with the PCR array’s findings.

### Both 6-BT and ATRA can attenuate the canonical Wnt signaling pathway and induce differentiation of HL-60 and primary AML blasts

**Figure 3:**
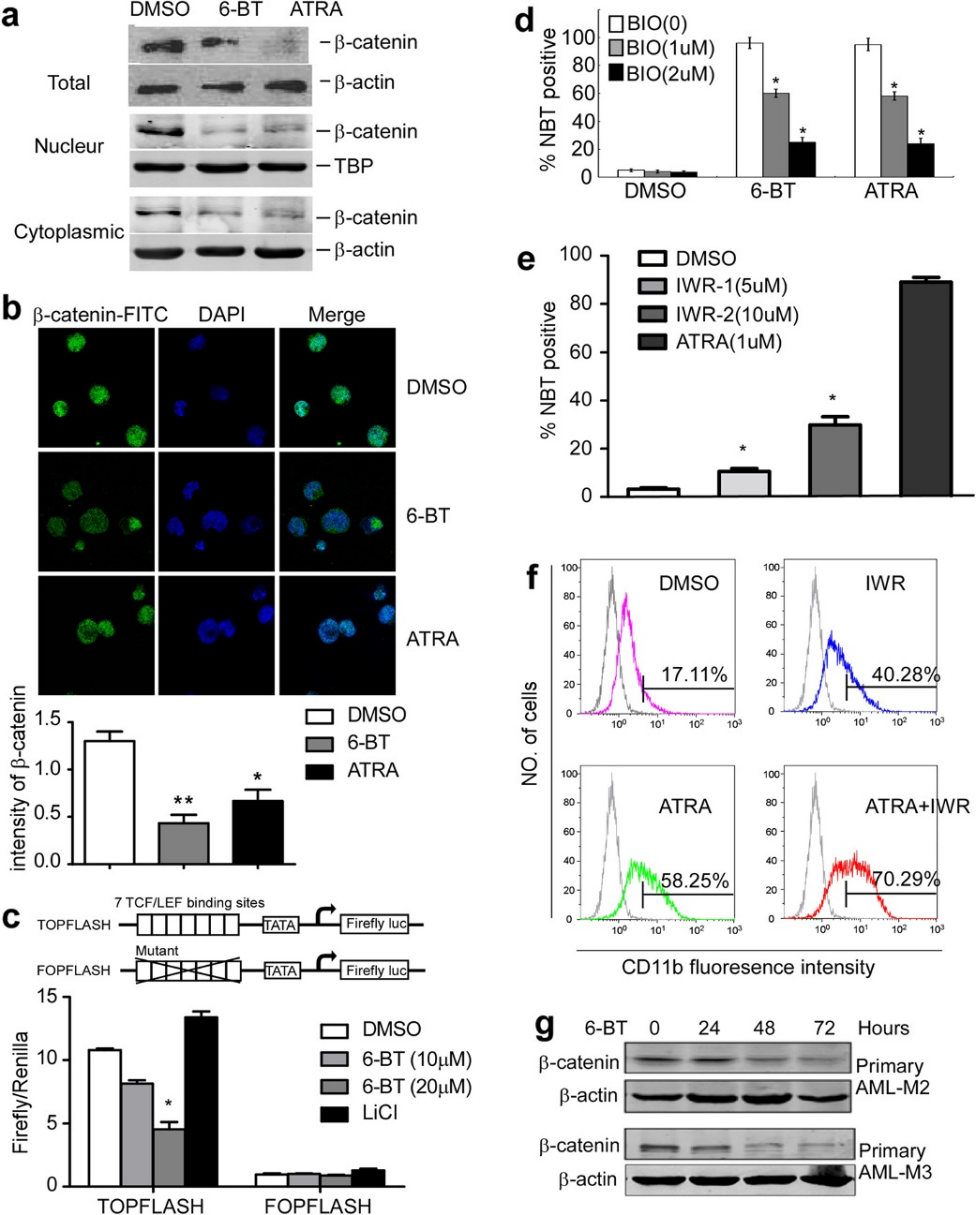
**Reduced activity of canonical Wnt signaling upon 6-BT and ATRA treatment. a**. Westernblot analysis showed that β-catenin expression in HL-60 cells was reduced by both 6-BT and ATRA treatment. β-actin as an endogenous control. Westernblot analysis of β-catenin in subcellular fractions in HL-60 cells after treatment by DMSO (0.01%), 6-BT (10 μM) or ATRA (1 μM) for 3 days. β-actin was used as cytoplasmic control and TBP was used as nuclear protein loading control. **b**. HL-60 cells were treated with DMSO (0.01%), 6-BT (10 μM) or ATRA (1 μM) for 3 days and then were fixed in 4% formaldehyde/PBS and permeabilized with 0.5% Triton X-100. β-catenin was visualized by immunofluorescence (green, left panel). The DNA-intercalating dye DAPI was used to identify cell nuclei (blue, center panel). The right panel presents a merged image to highlight the nuclear pool of β-catenin. **c**. Histogram showing TOPFLASH and FOPFLASH activation in HL-60 cells treated with DMSO, 6-BT or LiCl. *P <0.05. **d**. NBT reduction assay showed that BIO, which could up-regulate β-catenin protein, could partly abolish 6-BT- or ATRA-induced differentiation of HL-60 cells. Data are from three independent experiments. *P <0.05. **e**. HL-60 cells were treated with DMSO, IWR-1 (5 μM and 10 μM) and ATRA (1 μM,as a positive control) for 3 days, differentiation was assessed by NBT reduction. Data are from three independent experiments. *P <0.05. **f**. HL-60 cells were treated for 3 days with IWR-1 (10 μM), ATRA (0.5 μM) or both and then the cells were stained with anti-CD11b antibodies and analyzed by fluorescence-activated cell sorting (FACS). **g**. Westernblot analysis showed reduced expression of β-catenin in primary AML-M3 and AML-M2 leukemic blasts treated with 6-BT (10 μM) for 24, 48 or 72 hours.

Because the 6-BT induced HL-60 differentiation resulted in down-regulation of the molecules in the canonical Wnt signaling pathway, we then explored the underlying mechanisms of canonical Wnt signaling pathway related to the 6-BT induced HL-60 differentiation. β-catenin is the central molecule in the canonical Wnt signaling pathway, and its expression level and nuclear translocation can be used to assess the activity of this pathway [20]. We used ATRA, a well known differentiation-inducing agent, as a positive control in our experiment. After HL-60 was treated with 6-BT (10 μM) or ATRA (1 μM) for 3 days, we found that total β-catenin protein level was significantly decreased. Westernblot analysis of subcellular fractions confirmed that β-catenin was both decreased in the nucleus and cytoplasm of HL-60 cells (Figure 3a). To make the localization of β-catenin clear, we investigated the subcellular localization of β-catenin by immunofluorescence. We found β-catenin was located primarily in the nucleus and slightly in cytoplasm of vehicle (DMSO)-treated HL-60 cells indicating that the canonical Wnt signaling was constitutively activated in HL-60 cells. After treated with 6-BT and ATRA for 3 days, the amount of β-catenin was markedly decreased in HL-60 cells in both nucleus and cytoplasm (Figure 3b). Therefore, both 6-BT and ATRA repressed canonical Wnt signaling in HL-60 cells.

When β-catenin migrates to the nucleus, it acts as a co-stimulatory protein for the TCF/LEF family of transcription factors [21]. A promoter-reporter assay was performed using the β-catenin-responsive promoter TOPFLASH and the mutant control FOPFLASH [4]. TOPFLASH or FOPFLASH reporter plasmids were transfected into HL-60 cells, then incubated with DMSO, 6-BT or LiCl (positive control). TCF/LEF reporter activity was measured by luciferase assay. Luciferase activity of TOPFLASH significantly decreased after 6-BT treatment (Figure 3c).

GSK-3β phosphorylates and degrades β-catenin that results in the inhibition of the canonical Wnt signaling [22]. We tested whether BIO, a GSK-3β specific inhibitor, could activate canonical Wnt signaling and thereby inhibit 6-BT- and ATRA-induced differentiation of HL-60 cells. We first treated HL-60 cells with 6-BT or ATRA for 1 day, and then various concentration of BIO was added to the cells for another 2 days. NBT reduction assay was performed to determine the differentiation status of HL-60 cells. As seen in Figure 3d, BIO at a concentration of 2 μM significantly inhibited 6-BT- or ATRA-induced HL-60 differentiation by almost 80%.

We did more experiments to examine whether HL-60 cells differentiation can be induced by the addition of Wnt signaling inhibitor IWR-1 [23]. By NBT reduction assay, we observed that IWR-1 induced differentiation of the HL-60 cells (Figure 3e). IWR-1 also enhanced expression of CD11b, a widely known marker of granulocytic differentiation (Figure 3f). More important, we found that IWR-1 enhanced differentiation induced by ATRA (Figure 3g). Gandillet et al. noted that silencing of β-catenin, using a short hairpin RNA (shRNA) lentiviral approach, was the strikingly enhanced myeloid differentiation of the HL-60 cell line after ATRA induction [8], which was consistent with our results.

In order to confirm these results in HL-60 cells, we used primary leukemic blasts from AML patient marrows (AML-M3 and AML-M2 samples) that were treated with 6-BT (10 μM) for 24, 48 or 72 hours. Levels of β-catenin protein were measured by Western blot. β-catenin protein was significantly decreased upon treatment with 6-BT in all primary AML FAB M2 and M3 samples (Figure 3h). Taking these results together, we demonstrated that 6-BT- or ATRA-induced differentiation of HL-60 cells was through attenuation of the canonical Wnt signaling pathway.

### 6-BT and ATRA -induced HL-60 differentiation is through activation of the Non-canonical Wnt/Ca^2+^ signaling pathway

**Figure 4:**
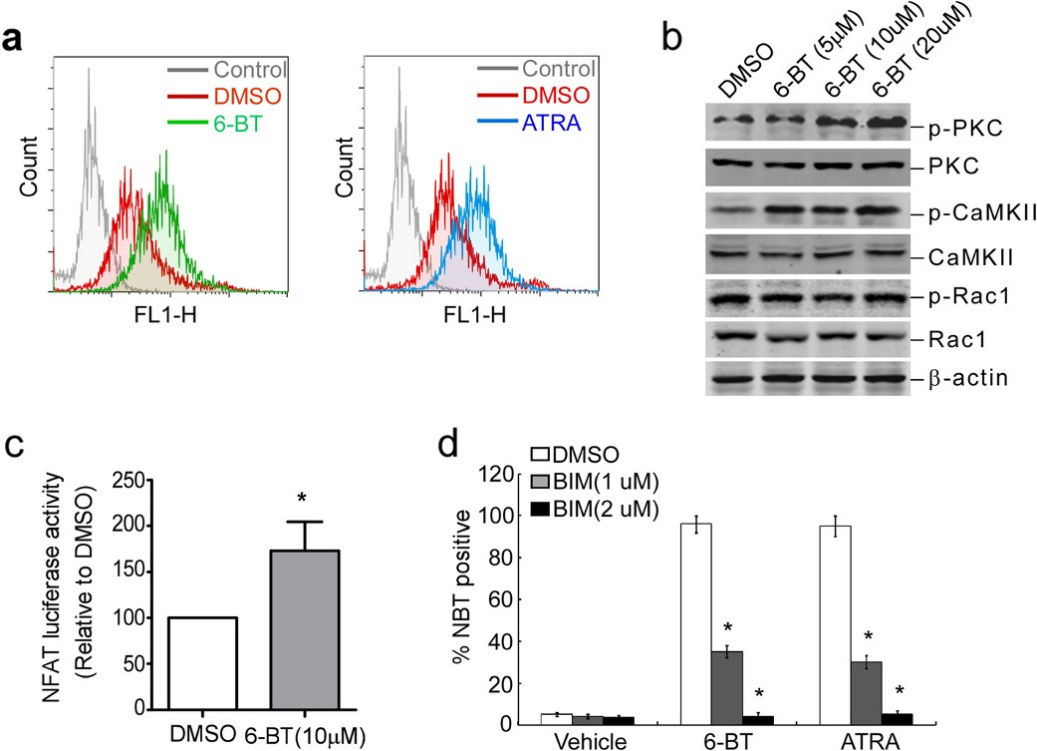
**Noncanonical Wnt/Ca** ^**2+**^ **signaling pathway is activated upon 6-BT or ATRA treatment. a**. HL-60 cells were labeled with Fluo-3/AM after treated with 6-BT (10 μM) or ATRA (1 μM) for 3 days and analyzed by fluorescence-activated cell sorting (FACS). **b**. After HL-60 cells were treated with DMSO (0.01%) or 6-BT (5, 10 or 20 μM) for 3 days, protein levels of PKC, p-PKC, CaMKII, p-CaMKII, Rac1 and p-Rac1 were analyzed by westernblot. **c**. Histogram showing NFAT luciferase activation in HL-60 cells treated with DMSO, 6-BT. *P <0.05. **d**. NBT reduction assay showed that the PKC inhibitor BIM abolished 6-BT- and ATRA-induced differentiation of HL-60 cells. Data are from three independent experiments. *P <0.05.

Since after 3 days of 6-BT treatment in HL-60 cells, we found significant fold increases in the transcriptional levels of the noncanonical Wnt ligands and receptors-Wnt5a (~12.12 fold increase), Wnt11 (~4.03 fold increase), FZD2 (~4.0 fold increase), FZD4 (~8.0 fold increase), FZD5 (~4.59 fold increase) and FZD7 (~7.46 fold increase) (Figure 1), we further investigated the role of non-canonical Wnt/Ca^2+^ pathway in HL-60 differentiation process induced by these agents. Firstly we detected intracellular Ca^2+^ concentration by Flou-3/AM after treatment by 6-BT and ATRA in HL-60 cells. Our results showed both 6-BT and ATRA could significantly elevate intracellular Ca^2+^ concentration in HL-60 cells (Figure 4a). To determine if the expression of downstream genes of noncanonical Wnt signaling pathway could be altered by 6-BT administration, we used westernblot analysis of p-CaMKII, p-PKC, and p-Rac1 after HL-60 cells were treated with 0, 5,10 and 20 uM 6-BT for 3 days. The phosphorylation levels of CaMKII and PKC were upregulated , while p-Rac1 and Rac1 did not changed (Figure 4b). Next we used the NFAT-luciferase construct, which contains NFAT binding sites, to determine the activity of the noncanonical Wnt signaling pathway. We detected a significant increase (66.3% increase) in NFAT-luciferase activity after 6-BT addition in HL-60 cells (Figure 4c). We pretreated HL-60 cells with the PKC inhibitor bisindoylmaleimide **(**BIM) for 4 hours and then tried to induce HL-60 differentiation by 6-BT (10 μM) or ATRA (1 μM) for 3 days, 6-BT and ATRA almost completely lost their capacity to induce differentiation of HL-60 cells (Figure 4d). These results strongly suggested that activation of the noncanonical Wnt/Ca^2+^ signaling was critical in 6-BT- and ATRA-induced differentiation of HL-60 cells (Additional file 1: Figure S1).

**Figure 5:**
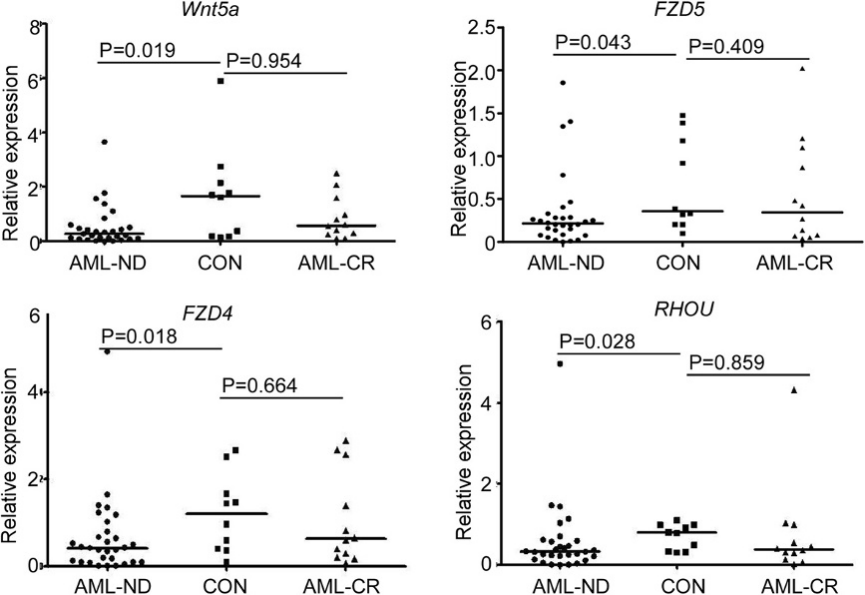
***Wnt5a*** **,** ***FZD*** **4,** ***FZD5*** **and** ***RHOU*** **are significantly down-regulated in newly diagnosed AML patients’ bone marrow samples compared to normal or CR group.** Expression levels of *Wnt5a*, *FZD4*, *FZD5* and *RHOU* were determined by RT-PCR analysis of bone marrow samples from AML patients, either newly-diagnosed (AML-ND) or in complete remission (AML-CR), and healthy donors (CON) using SYBR Green PCR Master Mix. The Mann–Whitney test was used to compare differences between the two groups.

We later examined the expression pattern of 4 genes, *FZD4, FZD5, Wnt5a* and *RHOU,* in the noncanonical Wnt/Ca^2+^ signaling pathway in primary AML cells from newly diagnosed AML patients (AML-ND), AML patients in complete remission (AML-CR) and normal controls (CON). The mean expression level of *FZD4, FZD5, Wnt5a* and *RHOU* were significantly down-regulated in AML-ND samples compared with normal controls (Figure 5). This result further suggested that deregulation of the Wnt signaling pathway was critical in the pathogenesis of AML.

## Discussion

The Wnt signaling pathway is involved in various processes, such as embryonic development, cell migration, proliferation, and differentiation [24, 25]. This pathway has also been extensively studied in tumorigenesis and shown to be involved in the development of several types of leukemia [26–28]. Activation of the canonical Wnt signaling pathway has been implicated recently in the pathogenesis of leukemia through maintenance of leukemic stem cells (LSCs) [29]. Overexpression of β-catenin is thought to be an independent adverse prognostic factor in AML [30, 31]. Accumulation of β-catenin in the nucleus has been demonstrated in both AML cell lines and primary blasts, indicating the activation of canonical Wnt signaling pathway [8, 32]. In a recent study, aberrant methylation of canonical Wnt antagonists was detected in four AML cell lines and in up to 64% of AML marrow samples [10, 33]. Yet, few studies have focused on canonical Wnt signaling pathway and differentiation of AML cells. In this study, we demonstrated that protein levels of β-catenin were down-regulated upon 6-BT and ATRA treatment. To explore the detailed mechanism of 6-BT induced decrement of β-catenin, the mRNA level of β-catenin was measured by qRT-PCR in HL-60 cells. As shown in Additional file 1: Figure S2, 6-BT treatment had little effect on the mRNA level of β-catenin, suggesting 6-BT elicited β-catenin protein attenuation could be mediated on post-translational level. we found that 6-BT treatment could upregulate the mRNA level of KREMEN1, which was a high-affinity DKK1 receptor that functionally cooperated with DKK1 to block Wnt/β-catenin signaling [34]. Exogenous addition of DKK1 inhibited both nuclear and cytoplasmic β-catenin level and decreased the nuclear/cytoplasmic ratio of β-catenin in a dosage-dependent manner [35, 36]. In conclusion, the downregulation of KREMEN1 might contribute to the effect of 6-BT on β-catenin. β-catenin is a cell-cycle regulated gene, so we detected cell cycle by FACS after HL-60 cells were treated with 0, 10, 20 μM 6-BT for 48 hrs. As shown in Additional file 1: Figure S3, 6-BT substantially increased the ratio of cells in the G0/G1 phase while concomitantly reduced the proportion of cells in the S phase in HL-60 cells. However, more studies were needed to clarify whether the cell-cycle effect is responsible for decrement of β-catenin afrer 6-BT treatment. Cell differentiation could also be induced by the addition of Wnt signaling inhibitor, such as IWR-1. Using GSK-3β inhibitor to enhance the canonical Wnt signaling pathway, 6-BT and ATRA lost their capacity in differentiation induction of HL-60 and primary leukemic cells, which suggested a critical role of this pathway in leukemic cell differentiation.

On the other hand, non-canonical Wnt/Ca^2+^ signaling is involved in activation of calcium/CaMKII [37] and PKC [13], which has been long considered as a tumor suppressor pathway [38]. Silencing Wnt5a, a non-canonical Wnt ligand, by methylation has been reported in some types of leukemia [15, 39]. Ectopic expression of Wnt5a resulted in inhibition of K562 cell growth and colony formation [15]. In our study, we showed that up-regulation of Wnt5a, elevation of intracellular Ca^2+^ concentration and up-regulation of p-PKC and p-CaMKII were observed in undergoing differentiation of leukemic cells after 6-BT or ATRA treatment. However, purified Wnt5a protein alone could not induce differentiation of HL-60 cells (data not shown). Amanda et al. detected ability of Wnt5a protein to directly stimulate intracellular calcium flux and found Wnt5a protein treatment at high dose did not alter the intracellular concentration of Ca^2+^ in 293 cells [40]. The activation of Wnt signaling by Wnt ligand is on receptor context. The downstream signaling events induced by Wnt5a also remain controversial and may be cell type- and receptor- dependent [41]. Based on this, Wnt5a protein alone may not sufficient to activate Wnt/Ca^2+^ signaling pathway. In our study, we found 6-BT up-regulated Wnt5a, as well as other noncanonical receptor molecules, such as FZD2, FZD4 and FZD5, indicating that receptor and/or co-receptor recruitment in Wnt/Ca^2+^/PKC activation is involved.

Previous studies reported the activation of noncanonical Wnt signaling lead to inactivation of canonical Wnt signaling. Topol and Ishitani et al. found activation of noncanonical Wnt signaling induced Ca^2+^ influx and promoted degradation of β-catenin independent of GSK3β and β-TRCP [42, 43]. Li et al. showed release of Ca^2+^ from cytosolic stores resulted in calpain-mediated degradation of β-catenin [44]. PKC-mediated β-catenin phosphorylation negatively regulated the Wnt/β-catenin pathway [45, 46]. Cho et al. reported that in adipocyte differentiation, the noncanonical Wnt signaling pathway inhibited the canonical Wnt signaling pathway, and BIM inhibited PKC that both can activate the Wnt/β-catenin signaling pathway [47]. Taken together, these results indicated that the downreglation of β-catenin could be elicited by Wnt/Ca^2+^ pathway. In our study, we discovered 6-BT and ATRA increased Wnt5a level, induced Ca^2+^ influx and upregulation of PKC, which might be the possible mechanism of β-catenin degradation. However, our PCR array data suggested that up-regulation of noncanonical and down-regulation of canonical Wnt signaling pathway seemed to happen simultaneously, raising some questions of Wnt signaling pathway in leukemogenesis. Does activation by treatment by 6-BT and ATRA of noncanonical Wnt signaling lead to inactivation of canonical Wnt signaling, or is this a coincidence? The interrelationship between canonical and noncanonical Wnt/Ca^2+^ signaling pathways needs further exploration. The Wnt signaling pathway is context-dependent transduced to both canonical and noncanonical pathways based on the expression profile of Wnt, sFRP, WIF, DKK, and FZD co-receptors and the activity of intracellular Wnt signaling regulators [40]. Besides Wnt5a, we observed the expression of other Wnt molecules changed upon 6-BT treatment. Does one particular Wnt molecule play a primary role in the differentiation process? By answering these questions, we may be able to identify precise targets for future development of AML differentiation therapy.

## Conclusions

We demonstrated that both canonical and noncanonical Wnt signaling pathways were collectively involved in the 6-BT and ATRA induced leukemic cell differentiation. *FZD4*, F*ZD5*, *Wnt5a* and *RHOU* are significantly down-regulated in bone marrow samples from newly diagnosed AML patients compared to normal controls, suggesting a critical role of Wnt signaling pathway in the pathogenesis of AML.

## Electronic supplementary material

Additional file 1: Figure S1: A proposed model of effect of 6-BT on both canonical and noncanonical Wnt signaling molecules in HL-60 cells. On the one hand, 6-BT treatment up-regulated level of KREMEN1 and down-regulated level of β-catenin, and concomitantly reduced the canonical Wnt signaling target gene *c-Myc*. On the other hand, 6-BT treatment also increased in the transcriptional levels of the noncanonical Wnt ligands and receptors-Wnt5a, Wnt11, FZD2, FZD4, FZD5 and FZD7. The phosphorylation levels of CaMKII and PKC, effecors in noncanonical Wnt pathway, were upregulated, indicating the activation of noncanonical Wnt signaling pathway. **Figure S2.** 6-BT treatment had little effect on the mRNA level of *β-catenin*. Real-time RT-PCR detected β-catenin mRNA levels in HL-60 cells after being treated with DMSO (0.01%) or 6-BT for 3 days. β-actin was used as control. The values represent the means ± S.E. (n = 3). **Figure S3.** 6-BT substantially increased the number of cells in the G0/G1 phase while concomitantly reduced the number of cells in the S phase in HL-60 cells. After being treated with DMSO (0.01%) or 6-BT for 2 days, HL-60 cells were were harvested and washed twice in PBS, then fixed in 75% alcohol over night at 4°C. After washed in cold PBS thrice, cells were resuspended in 1 mL PBS with 40 μg PI and 100 μg RNase A (Sigma-Aldrich, St Louis, MO) and incubated for 30 min at 37°C. Samples were then analyzed by FACS(Beckman, CA). (DOC 579 KB)
