# Supplementary material for: Wnt signaling is involved in 6-benzylthioinosine-induced AML cell differentiation
Source: BMC Cancer. 2014 Nov 27;14:886. doi: 10.1186/1471-2407-14-886 (PMC4289047; doi:10.1186/1471-2407-14-886)
Supplement: Supplementary file 1 — Additional file 1: Figure S1: A proposed model of effect of 6-BT on both canonical and noncanonical Wnt signaling molecules in HL-60 cells. On the one hand, 6-BT treatment up-regulated level of KREMEN1 and down-regulated level of β-catenin, and concomitantly reduced the canonical Wnt signaling target gene c-Myc. On the other hand, 6-BT treatment also increased in the transcriptional levels of the noncanonical Wnt ligands and receptors-Wnt5a, Wnt11, FZD2, FZD4, FZD5 and FZD7. The phosphorylation levels of CaMKII and PKC, effecors in noncanonical Wnt pathway, were upregulated, indicating the activation of noncanonical Wnt signaling pathway. Figure S2. 6-BT treatment had little effect on the mRNA level of β-catenin. Real-time RT-PCR detected β-catenin mRNA levels in HL-60 cells after being treated with DMSO (0.01%) or 6-BT for 3 days. β-actin was used as control. The values represent the means ± S.E. (n = 3). Figure S3. 6-BT substantially increased the number of cells in the G0/G1 phase while concomitantly reduced the number of cells in the S phase in HL-60 cells. After being treated with DMSO (0.01%) or 6-BT for 2 days, HL-60 cells were were harvested and washed twice in PBS, then fixed in 75% alcohol over night at 4°C. After washed in cold PBS thrice, cells were resuspended in 1 mL PBS with 40 μg PI and 100 μg RNase A (Sigma-Aldrich, St Louis, MO) and incubated for 30 min at 37°C. Samples were then analyzed by FACS(Beckman, CA). (DOC 579 KB) [file 12885_2014_5130_MOESM1_ESM.doc]

**Wnt signaling is involved in 6-benzylthioinosine-induced AML cell differentiation**

**Additional_file_1**


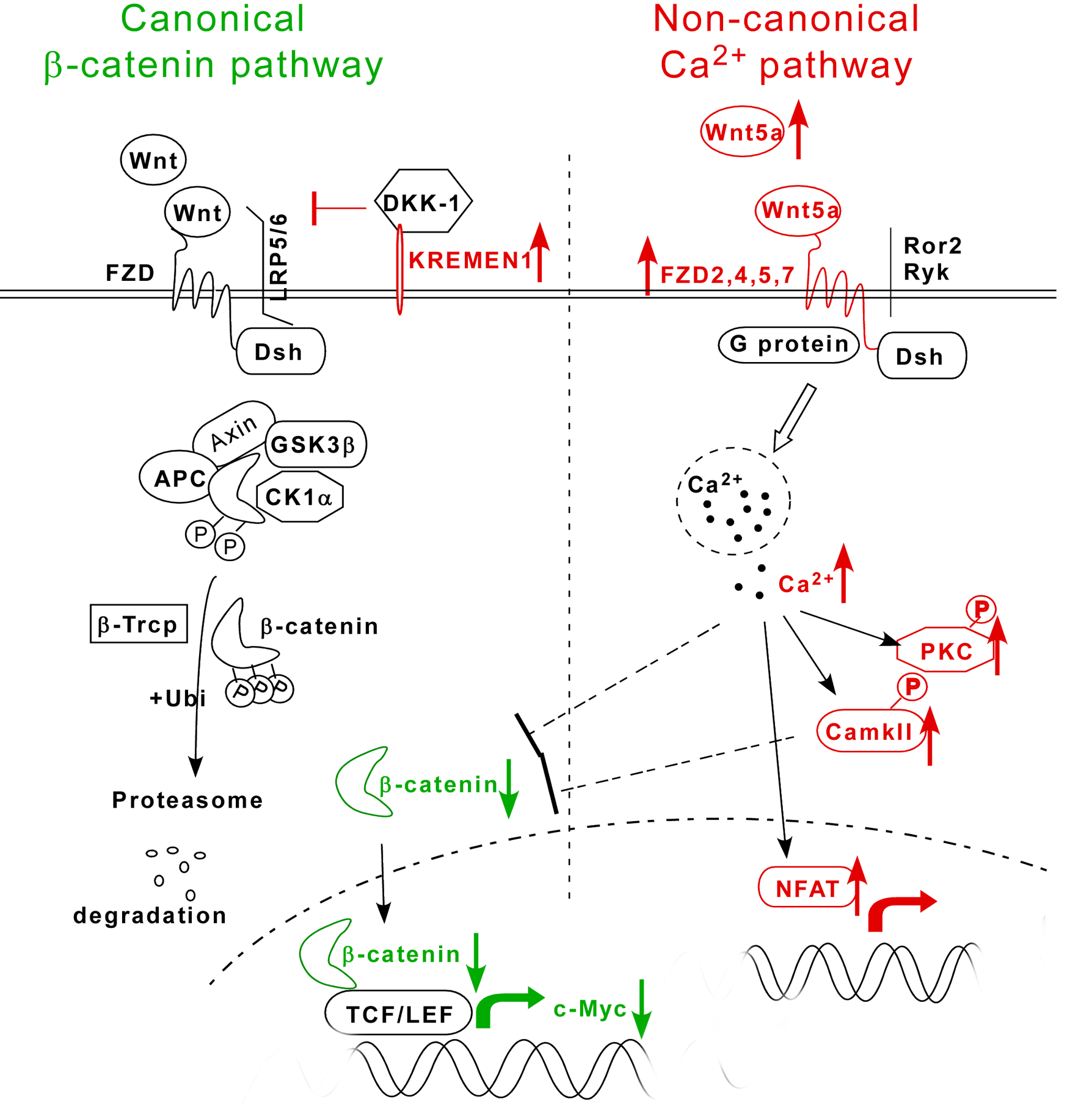


**Figure S1**. **A proposed model of effect of 6-BT on both canonical and noncanonical Wnt signaling molecules in HL-60 cells.** On the one hand, 6-BT treatment up-regulated level of KREMEN1 and down-regulated level of β-catenin, and concomitantly reduced the canonical Wnt signaling target gene *c-Myc*. On the other hand, 6-BT treatment also increased in the transcriptional levels of the noncanonical Wnt ligands and receptors-Wnt5a, Wnt11, FZD2, FZD4, FZD5 and FZD7. The phosphorylation levels of CaMKII and PKC, effecors in noncanonical Wnt pathway, were upregulated, indicating the activation of noncanonical Wnt signaling pathway.


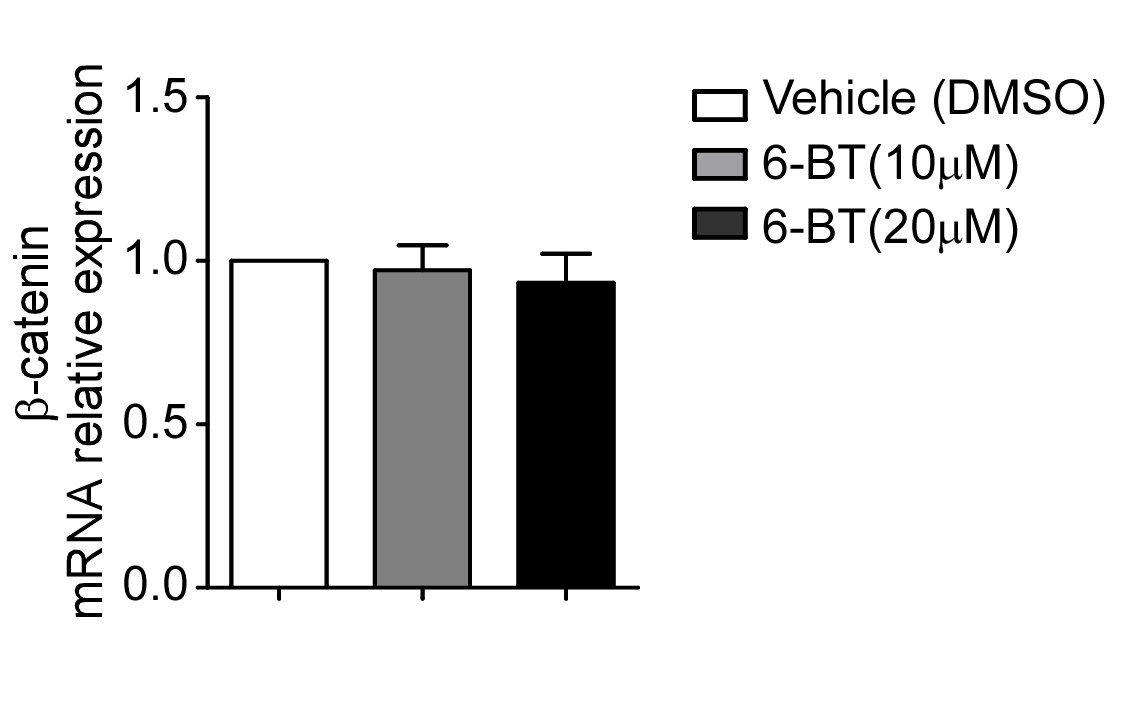


**Figure S2. 6-BT treatment had little effect on the mRNA level of *β-catenin*.** Real-time RT-PCR detected *β-catenin* mRNA levels in HL-60 cells after being treated with DMSO (0.01%) or 6-BT for 3 days. *β-actin* was used as control. The values represent the means ± S.E. (n = 3).


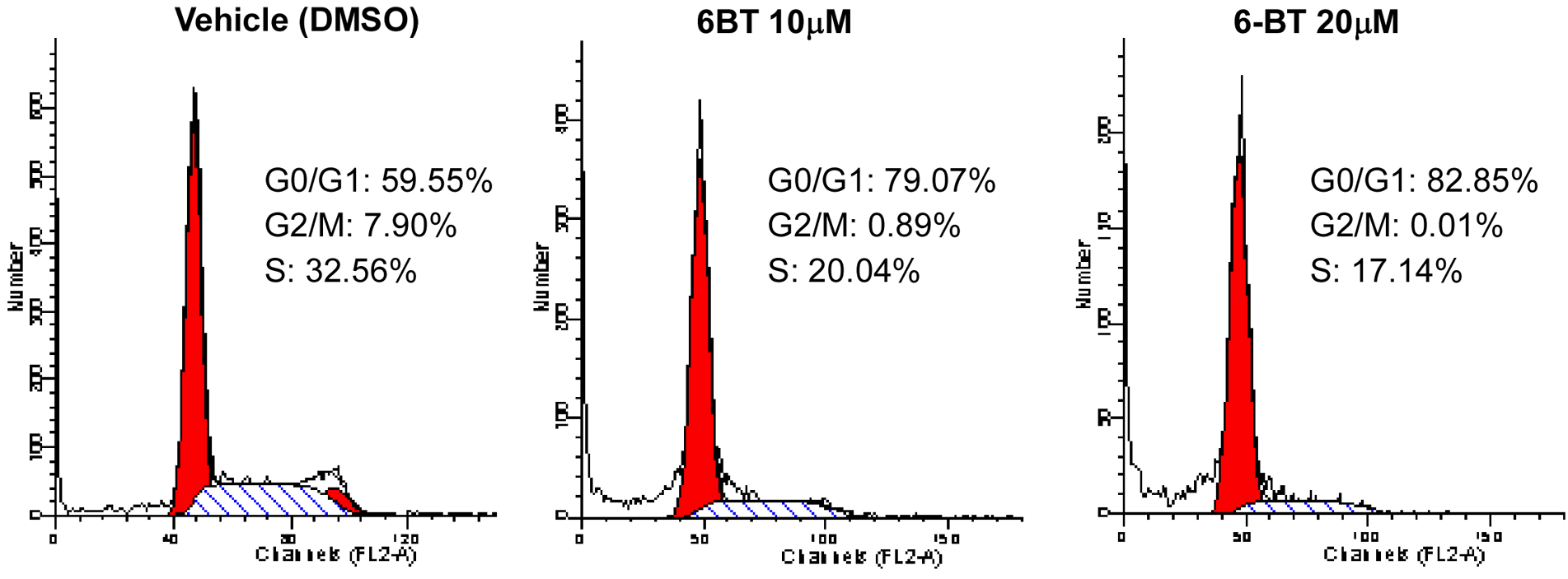


**Figure S3. 6-BT substantially increased the number of cells in the G0/G1 phase while concomitantly reduced the number of cells in the S phase in HL-60 cells.** After being treated with DMSO (0.01%) or 6-BT for 2 days, HL-60 cells were were harvested and washed twice in PBS, then fixed in 75% alcohol over night at 4°C. After washed in cold PBS thrice, cells were resuspended in 1 mL PBS with 40 µg PI and 100 µg RNase A (Sigma-Aldrich, St Louis, MO) and incubated for 30 min at 37°C. Samples were then analyzed by FACS(Beckman, CA)
